# Sudden quadriparesis after non-overdose local anesthesia

**DOI:** 10.1186/s12245-022-00423-7

**Published:** 2022-05-17

**Authors:** Wei-Chen Chen, Hsien-Yi Chen, Te-I. Weng, Chun-Kuei Chen

**Affiliations:** 1 Department of Emergency Medicine, Chang Gung Memorial Hospital, Linkou, Taoyuan Taiwan; 2 College of Medicine, Chang Gung University, Taoyuan, Taiwan; 3 Department of Emergency Medicine, National Taiwan University College of Medicine and National Taiwan University Hospital, Taipei, Taiwan; 4 Forensic and Clinical Toxicology Center, College of Medicine, National Taiwan University Hospital, National. Taiwan University, Taipei, Taiwan; 5 Institute of Forensic Medicine, College of Medicine, National Taiwan University, Taipei, Taiwan

**Keywords:** Local anesthetic systemic toxicity, Vocal cord cyst-removal surgery, Lipid emulsion, Quadriparesis

## Abstract

**Background:**

A well-known anesthetic, lidocaine is the most widely used local anesthetic. Local anesthetic systemic toxicity (LAST) is a life-threatening event with common and prominent presentations of central nervous system (CNS) toxicity and cardiovascular toxicity. The most frequent and prominent early warning signs and symptoms of LAST are central nervous system symptoms. While rare, cases quadriparesis after the administration of lidocaine has been reported.

**Case presentation:**

In this paper, we report a very rare case of quadriparesis after local anesthesia administration for vocal cord cyst-removal surgery, which dramatically improved after treatment. LAST can occur during various routes of lidocaine administration, such as local spray. A possible mechanism of our case could be the local diffusion of lidocaine to the spinal cord, which caused the symptoms to mimic anterior cord syndrome.

**Conclusions:**

Our case presented a favorable outcome following the administration of intravenous lipid emulsion (ILE) for non-over dose local anesthetic drug induced spinal cord inhibition symptoms. These findings highlight the need for further research on the use of ILE to reverse LAST and other adverse effects of local anesthetics.

## Background

Local anesthetic systemic toxicity (LAST) is a life-threatening event [1, 2], and can occur after administration of anesthetic drugs through various routes [2–4]. The causes of LAST are multifactorial and its presentation can vary case-by-case [5] though, the most common and early presentations of LAST are central nervous system (CNS) toxicity (e.g., seizure 68%, agitation 11%, or loss of consciousness 7%) [6] and cardiovascular toxicity (e.g., malignant ventricular arrhythmias and cardiac arrest) [3, 5]. LAST should therefore be considered strongly when in the presence of acute onset neurological symptoms or a change in mental state after exposure to a local anesthetic agent [5]. Quadriparesis is rare and atypical for LAST, though there have been reported cases during the use of nerve-blocking techniques, such as epidurals, intradural anesthesia, and interscalene blocks [7–10]. The quadriparesis might be evoked by mechanisms different from those of typical cases of LAST. In this case report, we present a very rare case of quadriparesis after local anesthesia was administered prior to vocal cord cyst-removal surgery, which dramatically improved after treatment.

## Case report

A 42-year-old female patient with no significant medical history visited the emergency department (ED) due to the sudden onset of generalized weakness. The patient reported she had received surgery for a vocal cord cyst removal 30 min prior. After the operation, she had walked out of the operating room unassisted and then felt generalized weakness and malaise. As a result, her muscle power dramatically decreased and subsequently the patient could not walk or move unassisted.

She was sent to the ED for further evaluation of her sudden symptoms. Upon arrival, her Glasgow Coma Scale score was E4V5M6. Her vital signs were taken: body temperature of 36.8 °C; heart rate of 60 beats per minute; and respiratory rate of 20 breaths per minute, with a blood pressure of 102/59 mmHg. She had no complaints of headache, chest pain, or neck pain. Physical examination showed neither limited eye movement nor nystagmus. The patient had no tongue deviation or fasciculation. Her sensory tests (proprioception and vibratory sensation) results showed that her relevant senses were intact and symmetrical. However, her muscle power was at grade 2 of 5 for her bilateral upper limbs and at grade 1 of 5 for her bilateral lower limbs. The Babinski sign showed no bilateral response and an absence of the sensation of pain. Her laboratory data—including her potassium, magnesium and calcium levels—were unremarkable. Electrocardiography revealed a sinus rhythm without prolonged QT_C_. C-spine X-ray images were obtained and revealed no bony deformity or spur formation.

In the available operation notes, it was recorded that the patient had been administered an epinephrine and lidocaine spray as a nasal decongestant, and a 2% lidocaine (5–10 mL) injection into the larynx for gargling. Then, dexamethasone (0.6 mL) was injected into the area of the right vocal cyst. Upon arrival at the ED, the patient’s serum lidocaine level was 0.54 µg/mL.

Suspecting an atypical presentation of LAST, we administered a 20% fat emulsion bolus (75 mL, 1.5 mL/kg). After 30 min, the patient’s muscle power improved: her upper limb muscle power increased from grade 2 of 5 to grade 4 of 5and her lower limb muscle power increased from grade 1 of 5 to grade 5. Muscle power had returned to normal 60 min after the patient had received the fat emulsion. The patient was discharged two hours after arriving at the ED. No further similar episode occurred after discharge.

## Discussion

Lidocaine is likely the most widely used local anesthetic in the world [11]. The most frequent and prominent early symptoms of LAST are CNS symptoms [6, 12]. Consequently, as the serum anesthetic concentration increases, adverse cardiovascular symptoms can occur after initial CNS symptoms. The serum anesthetic concentrations at which adverse cardiovascular and CNS events occur are used to calculate the cardiovascular/CNS (CC) ratio.. However, isolated cardiovascular events caused by LAST are also possible. Among various local anesthetics, lidocaine has a higher CC ratio, with signs of cardiovascular system toxicity occur at substantially higher plasma anesthetic levels than signs of CNS toxicity, thus resulting CNS toxicity most often occurring first [12].

According to a previous study, it was reported that local anesthetics depress the CNS in a dose-dependent manner [13]. Evidence of lidocaine toxicity may commence at serum concentrations > 5 μg/mL [13, 14]. The typical symptoms of CNS toxicity are seizure, numbness, agitation, auditory/visual disturbance, and loss of consciousness [3, 13, 15].

In accordance with the guidelines developed by the American Academy of Dermatology in 2016, local anesthetics are safe, cases of LAST is rare, and local anesthetics are continuously recommend for surgical procedures [16–18]. In our case, the serum lidocaine level was below the toxic overdose threshold; however, the patient still presented with atypical CNS inhibition symptoms. LAST can occur after administration of local anesthetics via various routes, though administration through intravascular injection has the highest associated risk [19–21]. Other routes, such as the use of transpharyngeal spray, intrascalene injection, or intrathecal administration has been reported to cause LAST [7, 19, 22]. All these routes could cause typical LAST through circulation of lidocaine to the brain and heart.

However, according to our patient's data, there was low serum lidocaine levels were reported and quadriparesis is similar to anterior cord syndrome; therefore, the mechanism could be different. The possible mechanism could be the local diffusion of lidocaine to the spinal cord. Quadriparesis after local anesthesia injections proposed pathophysiological has been reported after the administration of interscalene brachial plexus blocks. Reina et al. [23, 24] reports that epidural fat may act as a reservoir of lipophilic substances, resulting in a slow spread from this epidural fat to the epidural or intradural space, producing a delayed blocking of the CNS. Since the mechanism and clinical presentation were not typical for LAST, this case may only be spinal cord inhibition.

Quadriparesis has been reported as caused by spinal compression, a complete spinal block, a subdural block, and a spinal cord transient ischemic attack. We excluded most of the described causes as our patient had no similar history or risk factors for spinal compression, and experienced total recovery within an hour after treatment. It is also not complete spinal block because the patient was cognizant and oriented, hemodynamically stable, and breathing spontaneously, and the appearance of symptoms did not occur immediately after the operation. A subdural block was unlikely due to the operation method. Spinal cord transient ischemic attack was excluded due to our patient not having any vascular risk factors [25] and no spinal cord infarction after follow-up.

Intravenous administration of a lipid emulsion has been suggested as a treatment for LAST [26, 27]. The possible mechanism of action could be the formation of a lipid sink or lipid shuttle [28, 29]. It is also widely applied in some liposoluble drug intoxications, with the efficacy of this treatment confirmed through a systemic review and meta-analysis [30]. Complications associated with the use of intravenous lipid emulsion therapy for local anesthetic toxicity are rare, but include interferences with laboratory testing, pancreatitis, acute respiratory distress syndrome, fat overload syndrome, hypertriglyceridemia, and deep vein thrombosis [31, 32].

Intravenous lipid emulsion (ILE) as a resuscitative drug in other circumstances, such as total spinal anesthesia are reported in several case reports [32, 33]. There have been cases of spinal cord inhibition successfully reversed by ILE. The rapid improvement in our patient’s quadriparesis suggested that there was a positive response to the lipid emulsion therapy. Her improvement could also have been due to the drug being naturally metabolized. Other case reports have stated that CNS symptoms may last over 72 h or longer (up to 9 days) without lipid emulsion therapy [7, 10]. However, the mechanism of ILE in the intrathecal is still unclear and there lacks clear evidence of this mechanism [32, 34].

## Conclusion

Lidocaine, along with other local anesthetics, is reasonably safe. However, LAST may occur after local anesthetic administration via various routes during any procedure and it may present as any CNS symptom. ILE therapy remains the only antidote for LAST and may be beneficial for reversing the clinical symptoms and hasten recovery. Our case presented a favorable outcome following the guidelines of administering of ILE for non-over dose local anesthetic drug induced spinal cord inhibition symptoms. These findings highlight the need to further explore the use of ILE to reverse LAST and other adverse effects of local anesthetics.

## Data Availability

The datasets used and/or analyzed during the current study are available from the corresponding author on reasonable request.

## References

[1] Vadi MG, Patel N, Stiegler MP (2014). Local anesthetic systemic toxicity after combined psoas compartment-sciatic nerve block: analysis of decision factors and diagnostic delay. Anesthesiology.

[2] Yaddanapudi S (2011). Prevention of local anesthetic systemic toxicity. J Anaesthesiol Clin Pharmacol.

[3] El-Boghdadly K, Pawa A, Chin KJ (2018). Local anesthetic systemic toxicity: current perspectives. Local Reg Anesth.

[4] Gitman M, Fettiplace MR, Weinberg GL, Neal JM, Barrington MJ (2019). local anesthetic systemic toxicity: a narrative literature review and clinical update on prevention, diagnosis, and management. Plast Reconstr Surg.

[5] El-Boghdadly K, Chin KJ (2016). Local anesthetic systemic toxicity: continuing professional development. Can J Anaesth.

[6] Di Gregorio G, Neal JM, Rosenquist RW, Weinberg GL (2010). Clinical presentation of local anesthetic systemic toxicity: a review of published cases, 1979 to 2009. Reg Anesth Pain Med.

[7] Arcas-Bellas JJ, Cassinello F, Cercos B, del Valle M, Leal V, Alvarez-Rementeria R (2009). Delayed quadriparesis after an interscalene brachial plexus block and general anesthesia: a differential diagnosis. Anesth Analg.

[8] Morwald EE, Zubizarreta N, Cozowicz C, Poeran J, Memtsoudis SG (2017). Incidence of local anesthetic systemic toxicity in orthopedic patients receiving peripheral nerve blocks. Reg Anesth Pain Med.

[9] Norris D, Klahsen A, Milne B (1996). Delayed bilateral spinal anaesthesia following interscalene brachial plexus block. Can J Anaesth.

[10] FAIEZAH K, TAN T. Local Anaesthesia Systemic Toxicity (LAST)–A Stroke Mimicker. Med & Health. 2019;14(1):266–9. 10.17576/MH.2019.1401.27

[11] De Hert S, De Baerdemaeker L, De Maeseneer M (2014). What the phlebologist should know about local anesthetics. Phlebology.

[12] Wolfe JW, Butterworth JF (2011). Local anesthetic systemic toxicity: update on mechanisms and treatment. Curr Opin Anaesthesiol.

[13] Becker DE, Reed KL. Essentials of local anesthetic pharmacology. Anesth Prog. 2006;53(3):98-108; quiz 109-10. 10.2344/0003-3006(2006)53[98:EOLAP]2.0.CO;2.

[14] Yamashita S, Sato S, Kakiuchi Y, Miyabe M, Yamaguchi H (2002). Lidocaine toxicity during frequent viscous lidocaine use for painful tongue ulcer. J Pain Symptom Manage.

[15] Ruha AM, Levine M (2014). Central nervous system toxicity. Emerg Med Clin North Am.

[16] Starling J, Thosani MK, Coldiron BM (2012). Determining the safety of office-based surgery: what 10 years of Florida data and 6 years of Alabama data reveal. Dermatol Surg.

[17] Kouba DJ, LoPiccolo MC, Alam M (2016). Guidelines for the use of local anesthesia in office-based dermatologic surgery. J Am Acad Dermatol.

[18] Cherobin A, Tavares GT (2020). Safety of local anesthetics. An Bras Dermatol.

[19] Mehra P, Caiazzo A, Maloney P. Lidocaine toxicity. Anesth Prog 1998;45(1):38–41. (https://www.ncbi.nlm.nih.gov/pubmed/9790008).

[20] Dickerson DM, Apfelbaum JL (2014). Local anesthetic systemic toxicity. Aesthet Surg J.

[21] Dillane D, Finucane BT (2010). Local anesthetic systemic toxicity. Can J Anaesth.

[22] Gitman M, Barrington MJ (2018). Local anesthetic systemic toxicity: a review of recent case reports and registries. Reg Anesth Pain Med.

[23] Reina MA, Pulido P, Castedo J, Villanueva MC, Lopez A, Sola RG. [Characteristics and distribution of normal human epidural fat]. Rev Esp Anestesiol Reanim 2006;53(6):363–72. (https://www.ncbi.nlm.nih.gov/pubmed/16910144).

[24] Reina MA, Villanueva MC, Machés F, Carrera A, López A, De Andrés JA. The ultrastructure of the human spinal nerve root cuff in the lumbar spine. Anesth Analg. 2008;106(1):339–44. 10.1213/01.ane.0000295803.31074.dc.

[25] English SW, Rabinstein AA, Flanagan EP, Zalewski NL. Spinal cord transient ischemic attack: insights from a series of spontaneous spinal cord infarction. Neurol Clin Prac. 2020;10(6):480–3. 10.1212/CPJ.0000000000000778.

[26] Rothschild L, Bern S, Oswald S, Weinberg G (2010). Intravenous lipid emulsion in clinical toxicology. Scand J Trauma Resusc Emerg Med.

[27] Ozcan MS, Weinberg G (2014). Intravenous lipid emulsion for the treatment of drug toxicity. J Intensive Care Med.

[28] Heinonen JA, Litonius E, Salmi T (2015). Intravenous lipid emulsion given to volunteers does not affect symptoms of lidocaine brain toxicity. Basic Clin Pharmacol Toxicol.

[29] Neal JM, Barrington MJ, Fettiplace MR (2018). The third american society of regional anesthesia and pain medicine practice advisory on local anesthetic systemic toxicity: executive summary 2017. Reg Anesth Pain Med.

[30] Ok SH, Hong JM, Lee SH, Sohn JT (2018). lipid emulsion for treating local anesthetic systemic toxicity. Int J Med Sci.

[31] Ciechanowicz S, Patil V (2012). Lipid emulsion for local anesthetic systemic toxicity. Anesthesiol Res Pract.

[32] Turner FN, Shih RD, Fishman I, Calello DP, Solano JJ (2019). Total spinal anesthesia following an interscalene block treated with intravenous lipid emulsion. Cureus.

[33] Dun-Chi Lin J, Sivanesan E, Horlocker TT, Missair A (2017). Two for one: a case report of intravenous lipid emulsion to treat local anesthetic systemic toxicity in term pregnancy. A A Case Rep.

[34] Eldor J, Nguyen T (2018). Lipid emulsion for local anesthesia reversal (LAR) after prolonged spinal/epidural anesthesia. Jor Health Sci Development.

